# *In Vitro* ADME and Preclinical Pharmacokinetics of Ulotaront, a TAAR1/5-HT_1A_ Receptor Agonist for the Treatment of Schizophrenia

**DOI:** 10.1007/s11095-022-03267-1

**Published:** 2022-04-28

**Authors:** Guangqing Xiao, Yu-Luan Chen, Nina Dedic, Linghong Xie, Kenneth S. Koblan, Gerald R. Galluppi

**Affiliations:** DMPK and Clinical Pharmacology, Sunovion Pharmaceuticals Inc., 84 Waterford Drive, Marlborough, MA 01752 USA

**Keywords:** blood–brain barrier, CYP2D6, drug-drug interactions, phenotyping, TAAR1, ulotaront

## Abstract

**Purpose:**

Ulotaront (SEP-363856) is a TAAR1 agonist with 5-HT_1A_ agonist activity currently in clinical development for the treatment of schizophrenia. The objectives of the current study were to characterize the *in vitro* ADME properties, preclinical PK, and to evaluate the DDI potential of ulotaront and its major metabolite SEP-383103.

**Methods:**

Solubility, permeability, plasma protein binding, CYP inhibition and induction, transporter inhibition and uptake studies were conducted *in vitro*. Phenotyping studies were conducted using recombinant human CYPs and FMOs, human liver microsomes and human liver homogenates. Preclinical plasma and brain pharmacokinetics were determined after a single intraperitoneal, intravenous, and oral administration of ulotaront.

**Results:**

Ulotaront is a compound of high solubility, high permeability, and low binding to plasma proteins. Ulotaront metabolism is mediated via both NADPH-dependent and NADPH-independent pathways, with CYP2D6 as the major metabolizing enzyme. Ulotaront is an inducer of CYP2B6, and an inhibitor of CYP2D6, OCT1 and OCT2, while SEP-383103 is neither a CYP inducer nor a potent inhibitor of CYPs and human transporters. Ulotaront exhibits rapid absorption, greater than 70% bioavailability, approximately 3.5 L/kg volume of distribution, 1.5-4 h half-life, 12-43 ml/min/kg clearance, and good penetration across the blood–brain barrier in preclinical species.

**Conclusions:**

Ulotaront has been designated as a BCS1 compound by US FDA. The ability of ulotaront to penetrate the blood–brain barrier for CNS targeting has been demonstrated in mice and rats. The potential for ulotaront and SEP-383103 to act as perpetrators of CYP and transporter-mediated DDIs is predicted to be remote.

**Supplementary Information:**

The online version contains supplementary material available at 10.1007/s11095-022-03267-1.

## INTRODUCTION

Ulotaront is a novel investigational drug currently in Phase 3 clinical development for the treatment of patient with schizophrenia for which ulotaront has received US FDA Breakthrough Therapy Designation. In preclinical studies, ulotaront has demonstrated broad efficacy in animal models of schizophrenia (relating to positive and negative symptoms), as well as in rodent assays that are sensitive to antidepressants (1–3). Unlike all marketed antipsychotic drugs, ulotaront does not exert its effects via blockade of dopamine D_2_ or serotonin 5-HT_2A_ receptors. Although the mechanism of action of ulotaront has not been fully elucidated, preclinical studies suggest that agonism at TAAR1 and 5-HT_1A_ receptors contributes to its efficacy (1). TAAR1 has emerged as a promising therapeutic target for several neuropsychiatric disorders given its ability to modulate monoaminergic and glutamatergic neurotransmission (4, 5). In particular, TAAR1 agonists have generated interest as potential treatments for schizophrenia and other psychoses due to TAAR1-mediated regulation of dopaminergic circuitry (4, 5).

In a 4-week, randomized, double-blind placebo-controlled Phase 2 clinical trial (NCT02969328), ulotaront (50 -75 mg/day, po, flexibly dosed) demonstrated efficacy in the treatment of patients with an acute exacerbation of schizophrenia, with continued improvement observed over a 6-month open-label extension study (NCT02970929) (6, 7). In addition, ulotaront was generally safe and well-tolerated over the acute and open-label extension studies, with a safety profile distinct from medications used to treat schizophrenia (8). Notably, treatment with ulotaront was not associated with extra pyramidal side effects or other movement disorders, supporting its lack of D_2_ receptor blockade (6–8). In addition, ulotaront had no clinically meaningful effects on weight, lipids, glycemic indices, prolactin, or ECG parameters (including no evidence for prolongation of the QTc interval), all hallmark side effects associated with the current class of antipsychotic drugs.

Population pharmacokinetic (PK) analysis showed that ulotaront was rapidly absorbed and quickly cleared from plasma after oral administration to subjects with schizophrenia (9). Ulotaront exposures increased dose-proportionally at therapeutic dose levels ranging from 25 to 100 mg q.d. (9). Multiple metabolites of ulotaront have been identified from preclinical and clinical *in vitro* and *in vivo* studies (10). There are no human unique metabolites. Of the metabolites identified, SEP-383103 was the only major metabolite detected in the plasma of both preclinical and clinical species.

The primary objective of the current study was to characterize the *in vitro* absorption, distribution, metabolism, and excretion (ADME) profile, and preclinical PK of ulotaront and its major metabolite SEP-383103. The contribution of nicotinamide adenine dinucleotide phosphate (NADPH)-dependent and NADPH-independent enzymes to the metabolism of ulotaront, as well as to the biotransformation from ulotaront to SEP-383013, were investigated. In addition, *in vitro* studies assessed whether ulotaront and SEP-383103 are substrates, inhibitors, or inducers of human cytochrome P450 (CYP) enzymes and transporters. Finally, the penetration of ulotaront and SEP-383103 across the rodent blood–brain barrier (BBB) was investigated. Results from the *in vitro* ADME and preclinical PK studies, as well as the potential of CYP and transporter-mediated DDIs involving ulotaront and SEP-383103 are discussed in this paper.

## METHODS

### Solubility

The study was conducted at Eurofins BioPharma Product Testing (Columbia, MO). Ulotaront solubility was evaluated as the free base concentration in buffers over a pH range from 1.2 to 6.8 at 37 °C. The buffers used were hydrochloric acid buffer for pH 1.2, acetate buffer for pH 4.5, and phosphate buffer for pH 6.8. Saturated solutions of ulotaront in various pH buffers were allowed to equilibrate using the shake-flask method for 24 h. The concentration and solubility profile of the resulting samples were determined using high-performance liquid chromatography (HPLC) assay.

### Plasma Protein Binding, and Red Blood Cell Partitioning

The studies were conducted at Huntingdon Life Sciences (Huntingdon, UK). Plasma and whole blood from pooled CD-1 mice, Sprague Dawley (SD) rats, Beagle dogs, Marmoset monkeys, and human healthy volunteers were used for the assays. Plasma protein binding was determined using the ultrafiltration method. ^14^C-ulotaront was incubated with plasma at 37 °C for 1 h. After the incubation, aliquots were sampled and centrifuged at 2,000 g for 5 min, and the concentrations were determined by radioactivity. For red blood cell partitioning, ^14^C-ulotaront was incubated with fresh whole blood at 37 °C for 1 h. Following the incubation, a portion of the whole blood was sampled for radioactivity measurement, a separate portion was taken for measurement of the packed cell volume, and the remainder was centrifuged to obtain plasma for radioactivity measurement. ^14^C-ulotaront was used for all protein binding assays and red blood cell partitioning assays. ^14^C-ulotaront concentrations for protein binding assays were 0.01 to 100 µg/mL, and ^14^C-ulotaront concentrations for red blood cell partitioning assays were 1 to 100 µg/mL.

### Permeability

Ulotaront permeability study was conducted at Corning (Woburn, MA) using the Caco-2 monolayer system. Ulotaront (1 to 100 µM) was added to the apical and basolateral chambers to determine the permeability in the apical to basolateral (A to B), and basolateral to apical (B to A) directional, respectively. Transport buffer was Hanks Balanced Salt Solution (HBSS) buffered with 10 mM HEPES (N-[2- Hydroxyethyl] piperazine-N’-[2-ethanesulfonic acid]), pH adjusted to 7.4 with NaOH. Receiver solution was prepared by adding 1% organic solvent to transport buffer. The monolayers were incubated on an orbital shaker at 37 °C, with ambient humidity and CO_2_ for the duration of the transport assay. Samples were collected after 45, 90, and 120 min incubations and concentrations were determined by liquid chromatography with tandem mass spectrometry (LC–MS/MS). SEP-383103 permeability study was conducted at Covance (Madison, Wisconsin) using the Caco-2 monolayer system. SEP-383103 (1 and 5 µM) was added to the apical and basolateral chambers to determine the permeability in the apical to basolateral (A to B), and basolateral to apical (B to A) directional, respectively. Samples were collected after a 120-min incubation at 37 °C, and concentrations were determined by LC–MS/MS. For both ulotaront and SEP-383103 studies, digoxin and estrone-3-sulfate were used as the probe substrates of P-glycoprotein (P-gp) and Breast Cancer Resistance Protein (BCRP), respectively.

### Hepatocyte and Liver Microsome Stability

Hepatocyte stability studies were conducted at Huntingdon Life Sciences (Huntingdon, UK) using cryopreserved hepatocytes from pooled CD-1 mice, SD rats, Beagle dogs, Cynomolgus monkeys, and humans. Ulotaront (1, 10 and 50 µM) was incubated with cryopreserved hepatocytes (1 × 10^6^ cells/mL) in Supplemented Williams’ Medium E containing fetal calf serum for 30 min, 1, 2 and 4 h in an orbital shaking water bath (set at 60 rpm) at 37 °C under an atmosphere of humidified 95% oxygen and 5% CO_2_. Samples were then homogenized by ultrasonic disruption. Ulotaront stability in human liver microsome (HLM) stability assays were conducted at Xenotech (Kansas City, Kansas). Ulotaront (1, 10 and 100 μM) was incubated for 240 min with HLM (1 mg protein/mL) in 0.2-mL incubation mixtures containing potassium phosphate buffer (50 mM, pH 7.4), MgCl_2 _(3 mM) and EDTA (1 mM, pH 7.4) fortified with an NADPH-generating system and uridine 5'-diphospho-glucuronic acid (UDPGA). The disappearance of ulotaront after incubation with hepatocytes and HLM was analyzed by LC–MS/MS.

### *In Vitro* Phenotyping

Ulotaront phenotyping studies were conducted at Xenotech (Kansas City, Kansas) using recombinant human metabolizing enzymes and HLM in 0.2-mL incubation mixtures containing potassium phosphate buffer (50 mM, pH 7.4), MgCl_2_ (3 mM) and EDTA (1 mM, pH 7.4) with and without cofactors (mixed solution of an NADPH-generating system and UDPGA). ^14^C-ulotaront (1, 10 and 100 µM) was incubated with recombinant human CYP enzymes (rCYP1A2, 2B6, 2C8, 2C9, 2C19, 2D6 and 3A4; 25 pmol/mL), and human Flavin-containing Monooxygenase (FMO) enzymes (rFMO1, rFMO3 and rFMO5; 1 mg protein/mL). After 120 min incubation at 37 °C, the disappearance of ulotaront and formation of metabolites was analyzed by LC–MS/MS and radioactivity. Incubations of ^14^C-ulotaront with control microsomes (from non-transfected insect cells expressed without cytochrome b_5_) were included in the experiment as negative controls for the recombinant FMO enzymes. Michaelis–Menten enzyme kinetic constants (K_m_ and V_max_) for ulotaront metabolism (as measured by substrate loss) by recombinant CYP2D6 were determined.

The *in vitro* metabolism of ulotaront was also conducted at Covance (Madison, Wisconsin) using HµRel^®^ System to assess the involvement of CYP2D6 and non-CYP enzymes in ulotaront metabolism. HμRel^®^ human hepatic co-cultures were purchased from HμRel® Corporation (North Brunswick, New Jersey). Ulotaront (2 µM) was incubated with HµRel^®^ hepatocytes at 37 °C in an atmosphere of 5% CO_2_ in the absence and presence of 1-aminobenzotriazole (1 mM), or quinidine (1 µM) for 1, 2, 4, 6, 8, 12, 24, and 48 h. Ulotaront disappearance after the incubation was analyzed by LC–MS/MS.

The metabolism of ulotaront and formation of metabolites SEP-363854 and SEP-383103 were further investigated at WuXi AppTec Laboratory Testing Division (Cranbury, New Jersey) using HLM and human liver homogenates. Following ulotaront incubation with HLM (1 mg/mL) or human liver homogenates (0.2 mg/mL) in incubation mixtures containing potassium phosphate buffer (50 mM, pH 7.4), MgCl_2_ (3 mM) and EDTA (1 mM, pH 7.4) in the absence and presence of NADPH, the disappearance of ulotaront and the formation of metabolites SEP-383103 and SEP-363854 were monitored using LC–MS/MS. In addition, the formation of metabolite SEP-383103 was monitored using LC–MS/MS following ulotaront incubation with human liver homogenates in the absence and presence of phenelzine (10 µM), a Monoamine Oxidase (MAO) inhibitor (11), or raloxifene (1 µM), an Aldehyde Oxidase (AO) inhibitor (12).

### CYP Inhibition

*In vitro* CYP inhibition assessment of ulotaront (0.1 to 100 µM) was conducted at Xenotech (Kansas City, Kansas) using HLM. The inhibition of CYP1A2, CYP2A6, CYP2B6, CYP2C8, CYP2C9, CYP2C19, CYP2D6, CYP2E1, CYP3A4/5 (using two different marker substrate reactions; testosterone 6β-hydroxylation and midazolam 1′-hydroxylation) and CYP4A11 was determined. *In vitro* CYP inhibition assessment of SEP-383103 (0.1 to 100 µM) was conducted at Corning (Woburn, Massachusetts) using HLM. The inhibition of CYP1A2, CYP2B6, CYP2C8, CYP2C9, CYP2C19, CYP2D6 and CYP3A4/5 (using two different marker substrate reactions; testosterone 6β-hydroxylation and midazolam 1′-hydroxylation) was determined. Ulotaront or SEP-383013 were incubated with HLM in reaction mixtures containing NADPH-regenerating system and specific probe substrates (at concentrations approximately equal to their apparent K_m_) of the tested CYPs in 100 mM potassium phosphate buffer (pH 7.4) at 37 °C. LC–MS/MS was used for the quantitation of the metabolites of the probe substrates. Samples were centrifuged to precipitate the proteins, and supernatants were stored at -20 °C for subsequent analyzing metabolites of the probe substrates by LC–MS/MS.

To evaluate time-dependent inhibition of CYP activity, ulotaront or SEP-383103 was preincubated with HLM without and with an NADPH-generating system for 30 min, respectively, prior to the incubation with the probe substrates. Known direct and time-dependent inhibitors of CYP enzymes were used as positive controls in all experiments (Table S1).

### CYP Induction

*In vitro* CYP induction assessment of ulotaront (0.3 to 250 μM) was conducted at Xenotech (Kansas City, Kansas), and the *in vitro* CYP induction assessment of SEP-383103 (0.3 to 100 μM) was conducted at Corning (Woburn, Massachusetts). Both studies used cryopreserved primary human hepatocytes to evaluate the induction of CYP1A2, CYP2B6 and CYP3A4/5. Ulotaront and SEP-383103 were incubated with human hepatocytes from 3 donors for three consecutive days. After the incubation, enzyme induction was determined by measuring changes in mRNA expression using reverse transcription polymerase chain reaction (RT-PCR) and by conducting in situ catalytic enzyme activity assays using specific probe substrates. Phenacetin, bupropion, midazolam and testosterone were used as the probe substrates of CYP1A2, CYP2B6 and CYP3A4/5, respectively. Omeprazole (50 μM), phenobarbital (750 to1000 μM) and rifampin (10 to 20 μM) were used as positive inducer controls of CYP1A2, CYP2B6 and CYP3A4/5, respectively. Cytotoxicity was evaluated by Lactate Dehydrogenase (LDH) release.

### Transporter Inhibition and Uptake

*In vitro* transporter studies of ulotaront were conducted at Solvo (H-6726 Szeged, Közép fasor 52, Hungary). The inhibition and uptake studies with human Multidrug And Toxin Extrusion 1 **(**MATE1), MATE2-K, Organic Anion Transporter 1 (OAT1), OAT3, Organic Anion Transporting Polypeptide 1B1 (OATP1B1), OATP1B3, Organic Cation Transporter 1 (OCT1), OCT2 were conducted using transfected HEK293 cell lines expressing the transporters; the uptake studies with human P-gp and BCRP were conducted using transfected MDCKII cell lines expressing the transporters, and the inhibition on human P-gp, BCRP and Bile Salt Export Pump (BSEP) were conducted using transfected member vesicles expressing the transporters. Uptake studies were conducted at 4 concentrations of ulotaront: 0.5, 1, 5, and 10 µM for MATE1, MATE2-K, OAT1, OAT3, OATP1B1, OATP1B3, OCT1, and OCT2; and 0.25, 0.5, 2.5 and 25 µM for P-gp and BCRP. The inhibition of ulotaront was tested at 2 concentrations: 10 and 100 µM for MATE1, MATE2K, OAT1, OAT3, and OCT2; 75 and 750 µM for OATP1B1, OATP1B3 and OCT1; and 25 and 250 µM for P-gp, BCRP, and BSEP. The IC_50_ on OCT1 and OCT2 was determined at concentrations ranging from 0.3 to 300 µM.

*In vitro* transporter studies of SEP-383103 were conducted at Covance (Madison, Wisconsin). The inhibition studies with human MATE1, MATE2-K, OAT1, OAT3, OATP1B1, OATP1B3, OCT1, OCT2 were conducted using transfected HEK293 cell lines expressing the transporters; the inhibition and substate studies with human P-gp and BCRP were conducted using Caco-2 monolayer system. The inhibition was tested at 2 concentrations: 15 and 50 µM for MATE1, MATE2K, OAT1, OAT3, and OCT2; 30 and 100 µM for OATP1B1, OATP1B3, OCT1, P-gp, and BCRP. The permeability of SEP-383103 across Caco-2 cell monolayers was determined at 1 and 5 µM.

Transporter prototypical inhibitors and probe substrates were used for both ulotaront and SEP-383103 studies (Table S2).

All *in vitro* solubility, protein binding, red blood cell partitioning, stability, phenotyping, CYP and transporter studies were conducted in triplicates.

### Preclinical PK

All animal studies were conducted in accordance with the institutional animal and care with federal regulations and were approved by the respective Institutional Animal Care and Use Committees.

### Penetration across Mouse and Rat BBB

The distribution of ulotaront and SEP-383103 to mouse brain was conducted at ChemPartner Co., Ltd. (Shanghai, China) after single intraperitoneal (IP) and oral administrations of ulotaront (1, 3, 10 mg/kg) to male C57BL/6 mice. The IP and oral dosing solutions were prepared in 0.9% Saline at a concentration of 0.1, 0.3, 1 mg/mL. The dosing volume was 10 mL/kg for all PK studies. Whole blood samples were collected by cardiac puncture at 0.083, 0.25, 0.5, 1, 2, 4, 8, 12 and 24 h post IP or oral dosing into test tubes containing K_2_EDTA. Blood samples were centrifuged at 4 °C at 2,000 g for 5 min to obtain plasma samples. After blood collection, a mid-line incision was made in the animals’ scalp and skin was retracted. The skull overlying the brain was removed, and the whole brain was collected, rinsed with cold saline, dried on filtrate paper, weighed, and snap frozen by placing into dry ice. Brain sample was homogenized with 3 volumes (v/w) of PBS and stored at approximately -70 °C until analysis. Ulotaront and SEP-383103 plasma and brain concentrations were determined using LC–MS/MS.

The distribution of ulotaront to rat brain was conducted at Medicilon/MPI Preclinical Research (Shanghai, China). Ulotaront was formulated in PBS (1.43 g/L Na_2_HPO_4_, 0.255 g/L KH_2_PO_4_, 0.21 g/L KCl and 8.04 g/L NaCl, pH 7), and dosed to male SD rats at 10 mg/kg via oral gavage. Blood samples were collected via the cardiac puncture into tubes containing K_2_EDTA at 0.083, 0.25, 0.5, 1, 2, 4, 8, 12 and 24 h post dose, and centrifuged to harvest plasma samples for PK analysis. The brain of each animal was collected immediately after the blood collection. The whole brain was harvested, excised, and rinsed by saline, dried by filter paper, and then placed into one tube per animal tissue. Plasma and brain samples were stored at -80 °C until ulotaront concentration determinization by LC–MS/MS. Prior to analysis, brain samples were homogenized with buffer at 1:3 ratio right before analysis.

### Preclinical Plasma PK

Ulotaront rat plasma PK was conducted at Huntingdon Life Sciences (East Millstone, New Jersey). This was a cross-over design with the same male SD rats dosed intravenously then dosed orally after a one week washout period. Ulotaront was formulated in isotonic 100 mM acetate buffer with 70 nM mannitol. A group of three male SD rats received intravenous bolus injection at a dose level of 10 mg/kg, and by oral gavage at a dose level of 50 mg/kg a week later. Blood samples were collected from each animal into tubes containing K_3_EDTA at 0.083, 0.25, 0.5, 1, 2, 4, 8, 12 and 24 h post dose. Plasma samples were obtained by centrifugation of collected blood and stored at -80 °C until ulotaront concentration determinization by LC–MS/MS.

Ulotaront dog plasma PK was conducted at Huntingdon Life Sciences (East Millstone, New Jersey). This was a cross-over design with the same 3 male Beagle dogs dosed orally and then, after one week washout period, dosed intravenously. Ulotaront was formulated in isotonic 100 mM acetate buffer with 70 nM mannitol. Three male beagle dogs were dosed once by oral gavage with 5 mg/kg ulotaront. After a one week washout period, the same 3 male beagle dogs were dosed once intravenously via bolus injection with 1 mg/kg of ulotaront. Blood samples were collected from each animal into tubes containing K_3_EDTA at 0.083, 0.25, 0.5, 1, 2, 4, 6, 8, 12, 18, 24, and 48 h post dose. Plasma samples were obtained by centrifugation of collected blood and stored at -80 °C until ulotaront concentration determinization by LC–MS/MS. At the end of the treatment period, all animals were returned to the in-house colony.

Ulotaront monkey plasma PK was conducted at Medicilon/MPI Preclinical Research (Shanghai, China). Ulotaront was formulated in PBS (1.43 g/L Na_2_HPO_4_, 0.255 g/L KH_2_PO_4_, 0.21 g/L KCl and 8.04 g/L NaCl, pH 7), and dosed to male Rhesus monkeys via a single intravenous injection or a single oral gavage at 5 mg/kg. Blood samples were collected via the cardiac puncture into tubes containing K_2_EDTA at 0.083, 0.25, 0.5, 1, 2, 4, 6, 8, 12, 18, and 24 h post dose. Plasma samples were obtained by centrifugation of collected blood and stored at -80 °C until ulotaront concentration determinization by LC–MS/MS. At the end of the treatment period, all animals were returned to the in-house colony.

All preclinical plasma and brain PK studies on ulotaront and the major metabolite SEP-383103 were conducted with three animals for each dose and route of administration of ulotaront. Ulotaront doses ranging from 1 to 5 mg/kg for dog and monkey PK were selected mainly to evaluate the PK performance. Higher doses for mouse and rat PK were selected to support both PK and pharmacology studies. PK analyses were performed using the non-compartmental model with Phoenix® WinNonlin™ software (version 6.3 to 8.2, Pharsight Corporation).

### Ulotaront and Metabolite Quantification

For all *in vitro* and *in vivo* studies discussed in this paper, the concentrations of ulotaront and metabolites SEP-383103 and SEP-363854 were determined using either radioactivity, or LC–MS/MS based methods. The LC–MS/MS methods were developed at each research site by using ulotaront, SEP-383103 and SEP-363854 standards. The assay methods are provided in supplemental section. In addition, a sensitive LC–MS/MS method for simultaneous quantification of ulotaront and metabolite SEP-363854 was developed and validated in support of clinical studies (10).

### Data Analysis

#### Hepatic Clearance Calculation

Hepatic clearance (CL_hep_) is calculated using the well-stirred model based on intrinsic clearance (CL_int_) determine from *in vitro* hepatocyte stability assays:

$${\mathrm{CL}}_{\mathrm{hep}}=({\mathrm{Q}}_{\mathrm{h}}\times {\mathrm{f}}_{\mathrm{u}}\times {\mathrm{CL}}_{\mathrm{int}}/\mathrm{RBC})/({\mathrm{Q}}_{\mathrm{h}}+{\mathrm{f}}_{\mathrm{u}}\times {\mathrm{CL}}_{\mathrm{int}}/\mathrm{RBC})$$

Where f_u_ is ulotaront unbound fraction in preclinical and clinical plasma determined *in vitro.*

RBC is ulotaront red blood cell partitioning in preclinical and clinical species determined *in vitro.* Q_h_ is the hepatic blood flow in preclinical and clinical species (13).

#### Red Blood Cell Partitioning

Ulotaront blood to plasma concentration ratio (K_B/PL_) is calculated from the expression:

$${\mathrm{K}}_{\mathrm{B}/\mathrm{PL}}={\mathrm{C}}_{\mathrm{B}}/{\mathrm{C}}_{\mathrm{PL}}={\mathrm{K}}_{\mathrm{RBC}/\mathrm{PL}}\times\mathrm{H}+(1-\mathrm{H})$$

where C_B_ and C_PL_ represent the concentrations in whole blood and plasma, respectively. K_RBC/PL_ is the red blood cell (RBC) to plasma concentration ratio, and H is the volume of red blood cells to the total volume of whole blood (hematocrit).

#### DDI Assessment

DDI potential with ulotaront as an inducer of CYP2B6, and a direct inhibitor of CYP2D6 was assessed using the static mechanistic model according to US FDA Guidance (14). Default value 1 was used for ulotaront fraction of absorbed after oral administration (F_a_), and the fraction available after intestinal metabolism (F_g_). The fraction of hepatic clearance mediated by CYP enzymes (f_m_), and the fraction available after intestinal metabolism (F_g_) of CYP2B6 and CYP2D6 probe substrates were based on the simulations using SimCYP (version 20).

## RESULTS

### Solubility

The aqueous solubility of ulotaront was determined at three pH values using the shake-flask method. The solubilities at 37 ^o^C determined at pH 1.2, 4.5 and 6.8 were 423.5 ± 1.8, 434.4 ± 0.9 and 437.7 ± 2.7 mg/mL, respectively.

### Plasma Protein Binding, and Red Blood Cell Partitioning

The binding to plasma proteins and the red blood cell partitioning coefficient of ulotaront were determined at concentrations of 1 to 100 µg/mL for preclinical species, and 0.01 to 100 µg/mL for humans, as described in the methods. The mean values are shown in Table S3. Ulotaront has low binding to animal and human plasma proteins with an unbound fraction greater than 78%. Ulotaront is not preferentially sequestered into animal or human red blood cells. The blood to plasma ratio is close to unity. In addition, neither the protein binding nor the red blood cell partitioning coefficient is concentration dependent.

### Hepatocyte and Liver Microsome Stability

Ulotaront *in vitro* stability was evaluated in mouse, rat, dog, monkey and human hepatocytes at concentrations of 1, 10 and 50 µM. The intrinsic clearance (CL_int_) and hepatic clearance (CL_hep_) are summarized in Table S3. Cynomolgus monkey hepatic clearance was calculated based on plasma protein binding and red blood cell partitioning determined for Marmoset monkeys. Ulotaront exhibited low to moderate hepatic clearance in mouse, rat, monkey, and human hepatocytes, but high hepatic clearance in dog hepatocytes. The hepatic clearance determined from mouse, rat, monkey, and human hepatocytes is equivalent to < 25% of hepatic blood flow in the corresponding species, however the clearance determined from dog hepatocytes is about 50% of the hepatic blood flow.

The *in vitro* stability of ulotaront was also determined in HLM at 1, 10 and 100 µM. Similar to the observed *in vitro* stability in human hepatocytes, ulotaront showed slow metabolism in HLM. After 240 min incubation with HLM, there was less than 30% disappearance of ulotaront at 1 µM, and essentially no loss at 10 and 100 µM (Figure S1).

### Permeability

Ulotaront exhibited high permeability and low efflux ratio across Caco-2 monolayers. The permeability was determined at concentrations from 1 to 100 µM for 45 to 120 min. Under the experimental conditions, the permeability in the A-B direction ranged from 22 × 10^–6^ to 27 × 10^–6^ cm/s, the permeability in the B-A direction ranged from 28 × 10^–6^ to 30 × 10^–6^ cm/s, and efflux ratio ranged from 1.1 to 1.3 (Table S4). Similar permeability and efflux ratio were observed across the test concentration range and over the incubation period.

In contrast, SEP-383103 (1 and 5 µM) exhibited much lower permeability across Caco-2 monolayers. At 1 μM test concentration, the mean permeability values in the A-B and B-A directions were 2.3 × 10^–6^ and 2.7 × 10^–6^ cm/sec, respectively, and the efflux ratio was 1.2. At 5 μM test concentration, the mean permeability values in the A-B and B-A directions were 1.3 × 10^–6^ and 2.4 × 10^–6^ cm/sec, respectively, and the efflux ratio was 1.9.

### Phenotyping

*In vitro* studies were conducted to investigate the major enzymes involved in ulotaront metabolism, as well as in the biotransformation from ulotaront to the metabolites.

**Fig. 1:**
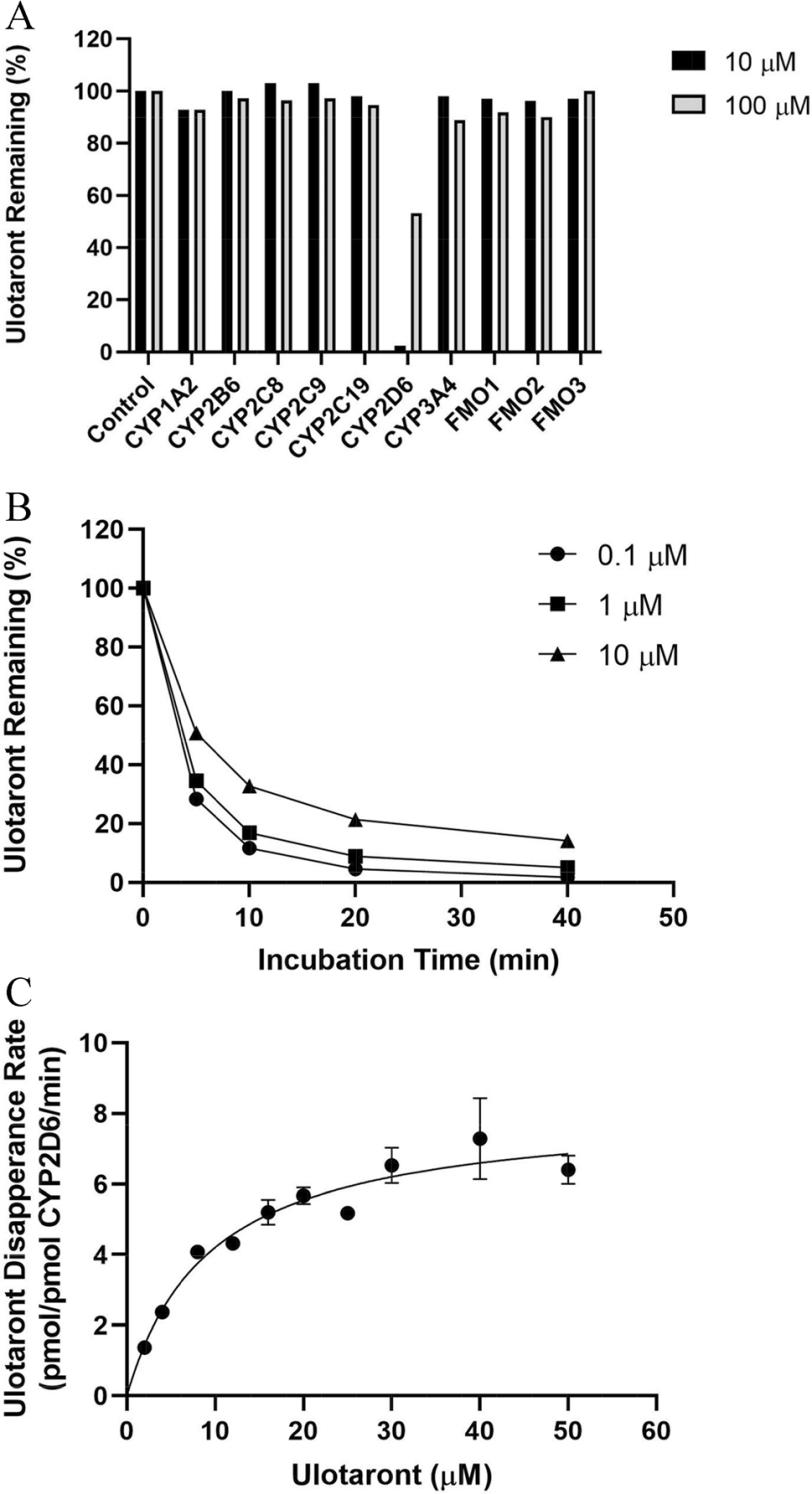
Phenotyping studies using recombinant enzymes. **A**: Disappearance of ^14^C-ulotaront (10 and 100 μM) following incubation with a panel of recombinant human CYP and FMO enzymes at 37 °C for 120 min. **B**: Disappearance of ulotaront (0.1, 1, and 10 μM) following incubation with recombinant CYP2D6 for 40 min. C: Kinetic study to determine the K_m_ and V_max_. Ulotaront was incubated with recombinant human CYP2D6 at 37 °C for 20 min. Data are presented as mean ± S.D.

Whether ulotaront metabolism was mediated by human CYP and FMO enzymes was investigated by analyzing parent disappearance after incubating ^14^C-ulotaront with a panel of recombinant CYP and FMO enzymes. Among the recombinant human CYP enzymes (rCYP1A2, 2B6, 2C8, 2C9, 2C19, 2D6 and 3A4) and FMO enzymes (rFMO1, FMO2 and FMO3) tested, significant loss of ulotaront was observed after the incubation with recombinant CYP2D6. Approximately 97% and 42% of ulotaront were metabolized after a 120-min incubation with recombinant CYP2D6 at 10 and 100 µM, respectively (Fig. 1A). More than 80% of ulotaront disappearance was observed over a 40 min incubation with recombinant CYP2D6 at lower concentrations (0.1, 1 and 10 µM) (Fig. 1B). The Michaelis–Menten enzyme kinetic constants (K_m_ and V_max_) for CYP-2D6-mediated metabolism were then determined as measured by ulotaront disappearance (Fig. 1C). The K_m_ was 4.84 ± 1.05 µM, and V_max_ was 7.42 ± 0.42 pmol/pmolCYP2D6/min.

In addition to monitoring ulotaront disappearance, the formation of metabolites after incubation with recombinant CYP and FMO enzymes was also analyzed by radioactivity. The relative abundance of metabolite formation is summarized in Table I, which indicates that the formation of the metabolites is mainly determined by CYP2D6.

**Table I:** Relative Abundance of Metabolite Formed from Recombinant Human CYP and FMO Incubation

Metabolites	CYP1A2	CYP2B6	CYP2C8	CYP2C9	CYP2C19	CYP2D6	CYP3A4	FMO1	FMO2	FMO3
SEP-363854	+	+			+	+ +				
M1						+ + +		+		
M2						+ + +				
M4						+ +	+			
M6						+ + +				
M7						+ + +				
M10	+ +					+ +		+ +		
M11						+ + +				
M12	+			+	+	+ +		+		

Relative abundance of metabolites is based on radioactivity

Because of the low metabolism from *in vitro* stability studies using HLM or human hepatocytes, ulotaront metabolism was also investigated by incubating ulotaront (2 µM) with HµRel^®^ hepatocytes for longer incubation. The impact of pan CYP inhibitor 1-aminobenzotriazole (1 mM) (15), and strong CYP2D6 inhibitor quinidine (1 µM) (16) on the metabolism was also examined to elucidate the role of non-CYP enzymes in ulotaront metabolism. The study indicated that ulotaront was metabolically stable under the experimental conditions. Further incubation for up to 48 h only led to approximately 10% loss of ulotaront. Pre-treatment with 1-aminobenzotriazole and quinidine slightly reduced metabolism, which is consistent with the involvement of CYP2D6 in ulotaront metabolism as observed from studies using recombinant human CYP enzymes.

**Fig. 2:**
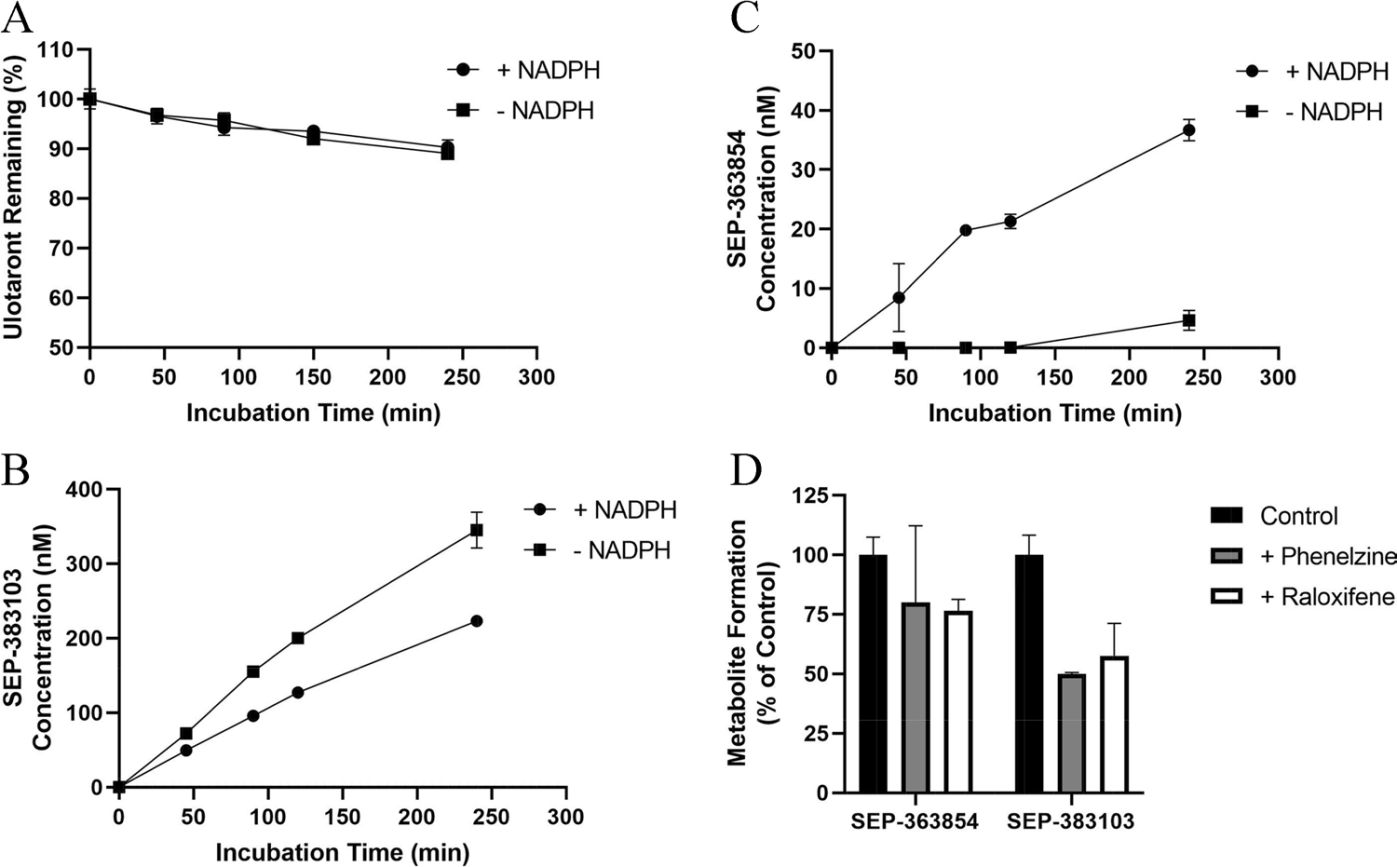
Phenotyping studies using human liver homogenates. **A**: Percentage remaining of ulotaront following incubation in the presence and absence of NADPH at 37 °C for 240 min at 0.1 µM. **B**: SEP-383103 formation after incubating ulotaront (10 μM) with human liver homogenates in the presence and absence of NADPH at 37 °C for 240 min. **C**: SEP-363854 formation after incubating ulotaront (10 μM) with human liver homogenates in the presence and absence of NADPH at 37 °C for 240 min. **D**: Formation of SEP-383103 and SEP-363854 after incubating ulotaront (10 μM) with human liver homogenates in the absence of NADPH, and the presence and absence of MAO inhibitor Phenelzine (10 µM), or Raloxifene (1 µM) at 37 °C for 240 min. Data are presented as mean ± S.D.

Multiple metabolites were identified in preclinical and clinical *in vitro* and *in vivo* studies, among them are SEP-383103 and SEP-363854. SEP-383103 is a major metabolite identified in the plasma of mice, rats, rabbits, dogs, monkeys, and humans after a single dose or repeat dose of ulotaront. SEP-363854 is identified as an abundant metabolite from *in vitro* studies, a major metabolite in the plasma of dogs and rabbits, but as a minor metabolite in the plasma of mice, rats, monkeys and humans. Using human liver homogenates, ulotaront metabolism and the biotransformation from ulotaront to metabolites SEP-383103 and SEP-363854 were investigated. Ulotaront was also metabolically stable *in vitro* even incubating with human liver homogenates at a low concentration (0.1 µM). There was less than 15% ulotaront disappearance after 240 min incubation, and ulotaront disappearance was similar in the presence of NADPH in comparison to in the absence of NADPH (Fig. 2A), suggesting NADPH-independent pathways are likely involved in ulotaront metabolism. Consistent with the role of NADPH-independent pathways in ulotaront metabolism, metabolite formation analyses showed while SEP-363854 was predominately formed in the presence of NADPH, more SEP-383103 was formed in the absence of NADPH than in the presence of NADPH (Fig. 2B and 2C). To identify the NADPH-independent enzymes that mediate the biotransformation, the formation of metabolites SEP-363854 and SEP-383103 was determined in the presence of phenelzine and raloxifene, inhibitors of NADPH-independent enzyme MAO (11), and NADPH-independent enzyme AO (12), respectively. After 240 min incubation of ulotaront with human liver homogenates, phenelzine (10 µM) and raloxifene (1 µM) inhibited SEP-383103 formation by 51% and 43%, respectively. Phenelzine (10 µM) and raloxifene (1 µM) also inhibited SEP-363854 formation by 20% and 24%, respectively (Fig. 2D).

### CYP Inhibition

The direct and time-dependent CYP inhibition by ulotaront and the major metabolite SEP-383103 were evaluated using HLM. Ulotaront and SEP-383103 concentrations were selected so that the highest test concentrations were at least 50-fold greater than the unbound C_max_ observed from clinical studies (9). At test concentrations ranging from 0.1 to 100 µM, ulotaront showed direct inhibition only on CYP2D6 with an IC_50_ value of 68 µM, but did not show direct inhibition on CYP1A2, CYP2A6, CYP2B6, CYP2C8, CYP2C9, CYP2C19, CYP2E1, or CYP3A4/5. Ulotaront showed similar inhibition on CYP2D6 after preincubating with HLM for 30 min without and with an NADPH-generating system, therefore ulotaront is not a time-dependent inhibitor of CYP2D6. In addition, there was no evidence of time-dependent inhibition on CYP1A2, CYP2A6, CYP2B6, CYP2C8, CYP2C9, CYP2C19, CYP2E1, or CYP3A4/5.

SEP-383103 did not show direct or time dependent inhibition on CYP1A2, CYP2B6, CYP2C8, CYP2C9, CYP2C19, or CYP3A4/5 at test concentrations ranging from 0.1 to 100 µM.

### CYP Induction

The potential to induce CYP1A2, CYP2B6, and CYP3A4/5 was investigated by incubating ulotaront and the major metabolite SEP-383103 with cryopreserved primary human hepatocytes from 3 donors for three consecutive days. Under the experimental conditions, ulotaront (0.3 to 250 μM) did not induce the mRNA expression or enzymatic activity of CYP1A2 or CYP3A4/5. Ulotaront did not induce CYP2B6 at concentrations less than 30 μM, but induced CYP2B6 mRNA expression and enzymatic activity of hepatocytes from two donors (lot HC10-23 and HC7-25) in a concentration dependent manner at concentrations ranging from 100 μM to 250 μM. Ulotaront induced CYP2B6 mRNA expression up to 6.48-fold and CYP2B6 enzymatic activity up to 7.19-fold, respectively. CYP2B6 induction parameters for mRNA expression and enzymatic activity are summarized in Table II.

**Table II:** CYP2B6 Induction by Ulotaront

Donor	HC10-23	HC10-23	HC7-25
Measurement	CYP2B6 mRNA	CYP2B6 enzyme activity	CYP2B6 enzyme activity
E_max_ (fold)	6.99 ± 0.12	7.34 ± 0.06	4.93 ± 0.12
EC_50_ (µM)	99.0 ± 3.6	83.3 ± 1.3	122 ± 6

Under the experimental conditions, SEP-383103 did not induce the mRNA expression or enzymatic activity of CYP1A2, CYP2B6, and CYP3A4/5 at concentrations ranging from 0.3 to 100 μM.

### Transporter Uptake and Inhibition

*In vitro* studies using transfected HEK293 cell lines or membrane vesicles were conducted to determine if ulotaront is a substrate or inhibitor of human efflux and uptake transporters. The transporters that were evaluated included efflux transporters P-gp, BCRP, and BSEP; and uptake transporters MATE1, MATE2-K, OAT1, OAT3, OCT1, OCT2, OATP1B1, and OATP1B3. The study indicated ulotaront is not a substrate of human P-gp or BCRP since the efflux ratios were less than 2, and the permeability and efflux ratio were not affected in the presence of the PSC833 and Ko143, the inhibitors of P-gp and BCRP (17, 18), respectively. Ulotaront is not a substrate of human MATE1, MATE2-K, OAT1, OAT3, OATP1B1, OATP1B3, OCT1, OCT2 either since there was less than twofold uptake to transfected cells/vesicles in comparison to mock cells/vesicles.

The inhibition potential of ulotaront on efflux and uptake transporters was tested at 2 concentrations as described in the methods. At the test concentrations, ulotaront did not show significant inhibition on P-gp, BCRP, BSEP, MATE1, MATE2K, OAT1, OAT3, OATP1B1 or OATP1B3, but exhibited inhibition of OCT1- and OCT2-mediated uptake of metformin, a probe substrate of OCT1 and OCT2 (19, 20). The inhibition was therefore further conducted at multiple concentrations to determine the IC_50_. The IC_50_ values for OCT1 and OCT2 are 18.37 μM, and 1.27 μM, respectively (Figure S2).

Whether SEP-383103 is an inhibitor of human P-gp, BCRP, MATE1, MATE2-K, OAT1, OAT3, OATP1B1, OATP1B3, OCT1, OCT2 was investigated at 2 concentrations as described in methods. Of the transporters evaluated, SEP-383103 only showed weak inhibition on OAT3 (~ 35% inhibition at 50 µM) and MATE1 (~ 20% inhibition at 50 µM).

### CYP-mediated DDI Assessment

Since ulotaront is an inducer of CYP2B6, and an inhibitor of CYP2D6, CYP-mediated DDI potential with ulotaront as the perpetrator was assessed using the static mechanistic model according to US FDA guidance (14). Bupropion and dextromethorphan were selected as the probe substrates of CYP2B6 and CYP2D6 (21–23), respectively. The fraction of bupropion hepatic clearance by CYP2B6 (f_m_), the fraction of dextromethorphan hepatic clearance by CYP2D6 (f_m_), and the fraction available of bupropion or dextromethorphan after intestinal metabolism (F_g_) were based on the simulations using SimCYP (version 20). Ulotaront maximum unbound hepatic inlet concentration ([I]_h_), and enterocyte concentration ([I]_g_) were based on the absorption rate k_a_ (0.99 h^−1^) and C_max_ (312 ng/mL at the intended dose of 100 mg) from clinical studies (9). The DDI assessments indicate concomitant ulotaront leads to 17% decrease in bupropion AUC, and 19% increase in dextromethorphan AUC (Table III).

**Table III:** Assessment of CYP-mediated DDI

	Victim			Perpetrator (ulotaront, 100 mg)					DDI
	Probe substrate	f_m_	F_g_	I_h_ (µM)	I_g_ (µM)	EC_50_ (µM)	E_max_ (fold)	K_i_ (µM)	AUCR
CYP2B6 Induction	Bupropion	0.41	0.99	5	29.3	83.3	7.34	NA	0.83
CYP2D6 Inhibition	Dextromethorphan	0.95	0.91	5	29.3	NA	NA	34	1.19

### Penetration across Mouse and Rat BBB

**Fig. 3:**
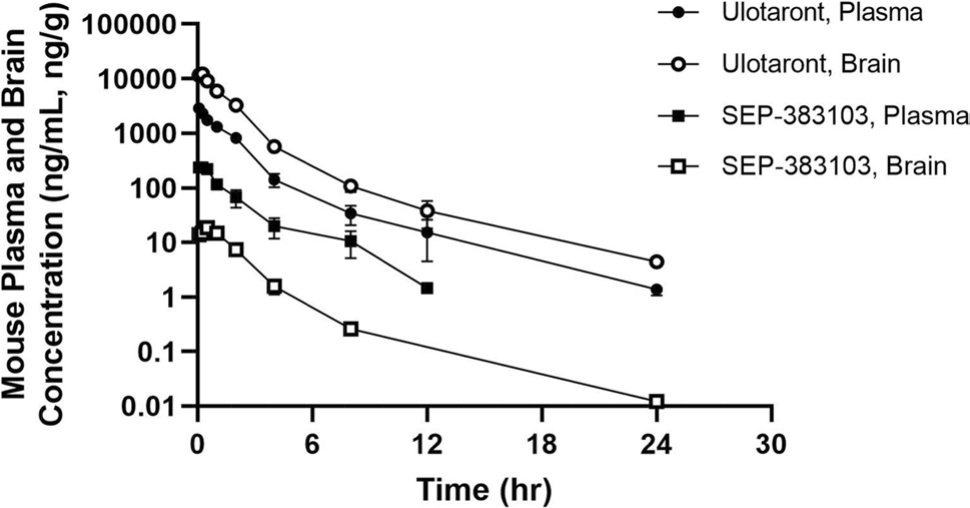
Ulotaront and SEP-383103 mouse plasma and brain PK profiles following a single IP dose of 10 mg/kg ulotaront. Concentrations are presented as mean ± S.D.

To evaluate the penetration across mouse BBB, ulotaront and SEP-383103 plasma and brain PK were determined following a single IP and oral administration of 10 mg/kg ulotaront to male C57BL/6 mice. Ulotaront was rapidly absorbed and distributed to mouse brain after a single IP and oral administration. Ulotaront brain maximum concentrations were reached within 30 min post dose, and brain exposures (C_max_ and AUC_inf_) were over fourfold greater than the exposures in plasma (Fig. 3, Figure S3, and Table IV). In contrast, SETP-383103 was barely distributed to the brain. SEP-383013 brain exposures (C_max_ and AUC_inf_) were at least tenfold lower than the plasma exposures (Fig. 3, Figure S3, and Table IV). The penetration of ulotaront and SEP-383103 across mouse BBB was also investigated following a single IP or oral administration of ulotaront at 1 mg/kg and 3 mg/kg, and similar results were observed.

**Table IV:** Ulotaront and SEP-383103 Plasma and Brain PK following a Single IP or PO Administration at 10 mg/kg of Ulotaront

Species	Route	Analyte	Tissue	T_1/2_	T_max_	C_max_		AUC_inf_	
				hr	hr	ng/mL, ng/g	B/P ratio	ng*hr/mL, ng*hr/g	B/P ratio
Mouse	IP	Ulotaront	Plasma	3.46	0.08	2857		4408	
			Brain	3.55	0.25	12,267	4.3	22,483	5.1
Mouse	IP	SEP-383103	Plasma	4.21	0.08	259		893	
			Brain	1.21	0.50	18.5	0.07	30.7	0.03
Mouse	PO	Ulotaront	Plasma	2.35	0.25	962		3668	
			Brain	2.70	0.25	5187	5.4	17,839	4.9
Mouse	PO	SEP-383103	Plasma	2.30	0.25	155		1373	
			Brain	11.60	0.25	11.88	0.08	60.2	0.04
Rat*	PO	Ulotaront	Plasma	2.10	0.25	1750		5352	
			CSF	2.03	0.25	1161	0.66	4544	0.85
			Brain	2.33	0.25	3762	2.1	16,854	3.1

^*^Partially reported in (1)

Ulotaront penetration across rat BBB was investigated following a single oral administration of 10 mg/kg ulotaront to male SD rats. Consistent with the observation from mouse brain distribution study, ulotaront was rapidly absorbed and distributed to brain after oral administration. Ulotaront brain maximum concentrations were reached within 30 min post dose. The brain exposures (C_max_ and AUC) were over twofold greater than the exposures in plasma, and the cerebrospinal fluid (CSF) exposures (C_max_ and AUC) were about 60–85% of the exposures in plasma (Table IV).

### Preclinical Plasma PK

**Fig. 4:**
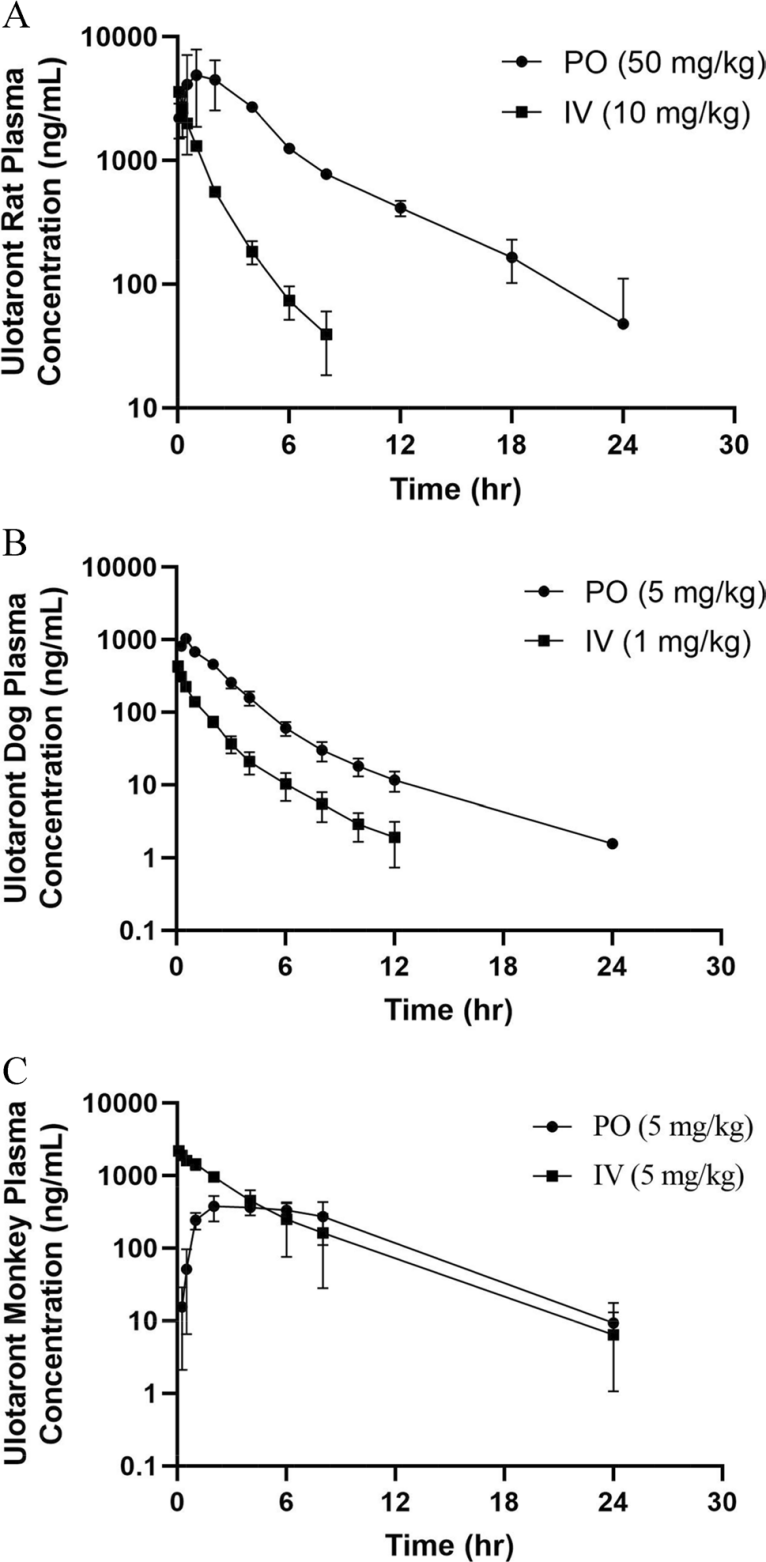
Ulotaront plasma PK profiles following a single intravenous and oral administration. **A**: Rat PK, **B**: Dog PK. **C**: Monkey PK. Concentrations are presented as mean ± S.D.

Ulotaront plasma pharmacokinetics in preclinical species were determined after a single oral and intravenous administration to male SD rats, male Beagle dogs and male Rhesus monkeys. The mean PK parameters are summarized in Table V, and concentration–time profiles are plotted in Figs. 4A-4C. Parts of the results from the rat and Rhesus monkey PK studies have been included in a previous publication (1). Following a single oral dose, ulotaront was rapidly absorbed with the maximum plasma concentrations reached in less than 2 h post dose (except 6 h in monkeys). Ulotaront showed high bioavailability (~ 100% in rats, 92% in dogs, and 71% in monkeys), and half-life between 3 to 4.1 h in preclinical species.

**Table V:** Ulotaront Preclinical Pharmacokinetics

Species	Route	Dose	T_1/2_	T_max_	C_max_	AUC_last_	V_ss_	fe	total CL	CLr	CL_h_	F
		mg/kg	hr	hr	ng/mL	ng*hr/mL	L/kg	%	mL/min/kg	mL/min/kg	mL/min/kg	%
Rat*	IV	10	1.6			4380	3.5	35.8	42.7	15.3	27.4	
Rat*	PO	50	3.4	2	5040	26,000						~ 100
Dog	IV	1	2.4			510	3.5	4.8	28.2	1.4	26.8	
Dog	PO	5	4.1	0.5	1030	2360						92
Monkey*	IV	5	3.1			6563	3.6	10	11.9	1.2	10.7	
Monkey*	PO	5	3	6	431	4708						71

^*^Partially reported in (1)

Following a single intravenous administration, ulotaront showed a volume of distribution of approximately 3.5 L/kg, and half-life between 1.6 to 3.1 h in rats, dogs and monkeys. Ulotaront exhibited clearance with less than 50% hepatic blood flow in rats and monkeys, but greater than 50% hepatic blood flow in dogs. The hepatic blood clearance determined in rats, dogs and monkeys after a single intravenous dose were 27.4, 26.8 and 10.7 mL/min/kg, equivalent approximately to 50%, 86% and 25% of hepatic blood flow in rats, dogs, and monkeys, respectively. The amount of ulotaront excreted in rat urine is equivalent to 35.8% of the administered dose. The amounts of ulotaront excreted in the urine of dogs and monkeys were significantly lower, representing 4.8% and 10% of the administered dose in dogs and monkeys, respectively.

## DISCUSSION

The *in vitro* ADME properties and preclinical pharmacokinetics of ulotaront have been characterized. Ulotaront solubility is not pH dependent and is more than 100-fold greater than the intended clinical dose in 250 mL (100 mg/250 mL = 0.4 mg/mL), the threshold for classification as a highly soluble drug within the test pH range from1.2 to 6.8 (24). These results demonstrate that ulotaront is highly soluble. The high solubility and high permeability (Table S4) are consistent with the high absorption rate observed from mass balance studies in humans using ^14^C-ulotaront (Data on file at Sunovion). Additionally, the low efflux ratio and high permeability are consistent with ulotaront’s ability to penetrate BBB, which has been demonstrated in mice and rats, and makes ulotaront ideal for CNS targeting.

The metabolic stability of ulotaront in preclinical species was determined *in vitro* using hepatocytes, and *in vivo* after a single dose via intravenous administration. Both *in vitro* and *in vivo* studies indicated low to moderate hepatic clearance in rats and monkeys and high hepatic clearance in dogs (Table IV and Table S3). The *in vivo* to *in vitro* ratio of hepatic clearance for rats, dogs and monkeys is 5.5, 1.7, and 1.3 respectively. Ulotaront elimination in humans is mostly via hepatic clearance, with renal clearance contributing approximately 15% of total clearance. The apparent total clearance observed from clinical studies is 32.5L/h (or 7.7 mL/min/kg based on a body weight of 70 kg) (9). Since ulotaront is projected to have good bioavailability (greater than 70%), the estimated ulotaront human *in vivo* hepatic blood clearance of 4.0 mL/min/kg [calculated as observed apparent total clearance (7.7 mL/min/kg) × estimated bioavailability (0.7) × fraction of total clearance due to metabolism (0.85) × 1/RBC] is likely within twofold of the hepatic clearance determined *in vitro* (3.9 mL/min/kg, Table S3). Therefore, based on the *in vivo* to *in vitro* ratios cited above, there is a good *in vitro and in vivo* correlation of hepatic clearance in dogs, monkeys, and humans, but not in rats, where the ratio was 5.5.

Phenotyping studies using recombinant human CYPs and FMOs indicated that the *in vitro* metabolism of ulotaront is mainly determined by CYP2D6, and to a much lesser extent by CYP1A2, CYP2C19 and FMO1 (Table I, Figs. 1A-1C). Studies using human liver homogenates suggest that both NADPH-dependent and NADPH-independent enzymes are involved in ulotaront metabolism (Fig. 2A), while the biotransformation of ulotaront to SEP-383103 is more determined by NADPH-independent enzymes, such as MAO and AO (Figs. 2B and 2D). Due to the low metabolism when studied *in vitro*, the fraction of ulotaront hepatic clearance (f_m_) by CYPs, FMOs, and NADPH-independent enzymes could not be confidently assigned just based on *in vitro* stability studies. By comparing the apparent clearance observed between subjects carrying CYP2D6 poor metabolizer (PM) and extensive metabolizer (EM) phenotypes from clinical studies, CYP2D6 f_m_ was estimated to be 0.53 (Data on file at Sunovion).

Ulotaront is a reversible inhibitor of CYP2D6 and an inducer of CYP2B6 (Table II). However, assessments using either the static mechanistic model (Table III) or PBPK modeling (Data on file at Sunovion) predict that concomitant ulotaront leads to less than 25% change in probe substrate exposures (e.g., 0.8 < AUCR < 1.25), suggesting CYP-mediated DDI potential with ulotaront as the perpetrator is remote.

Most of the inhibitors for renal transporters OCT2 and MATE1 or MATE2K are overlapped, although relatively specific inhibitors for MATE1 and MATE2K such as famotidine, nizatidine and pyrimethamine, and relatively specific inhibitors for OCT2 such as disopyramide, have been identified (25–29). In this paper, we report that ulotaront is an inhibitor of OCT2 but not MATE1 or MATE2K (Figure S2). PBPK simulation suggests concomitant ulotaront likely will not affect the PK of OCT2 substrates to a clinically meaningful extent (Data on file at Sunovion), although this remains to be evaluated and confirmed in an ongoing clinical DDI study.

In preclinical species, ulotaront exhibited rapid absorption, moderate volume of distribution, and high bioavailability (Table V). The high bioavailability in monkeys is consistent with the low hepatic clearance. However, the high bioavailability in rats and dogs was not consistent with the moderate to high clearance determined *in vitro* and *in vivo*. The reason for the disconnection is not known. After a single IP or oral dose, ulotaront was well distributed to mouse and rat brain, with the total brain exposures being over fourfold and twofold greater than the total plasma exposures in mice and rats, respectively (Table IV). Ulotaront’s ability to penetrate mouse and rat BBB is consistent with the high permeability and not subjected to P-gp or BCRP mediated efflux.

SEP-383103 is not an inhibitor nor is it an inducer of major human CYP enzymes. It is not a potent inhibitor of human transporters either. Although it showed inhibition of OAT3 and MATE1, the IC_50_ is at least 50-fold higher than SEP-383103 unbound C_max_ at steady state after repeat dose of 100 mg ulotaront. Therefore, the potential for SEP-383103 to be a perpetrator of CYP- and transporter-mediated DDI is remote. In contrast to ulotaront, SEP-383013 was barely distributed to mouse brain. The poor ability to penetrate mouse BBB may be partially explained by the low permeability. SEP-383103 demonstrated no significant activities in an *in vitro* broad panel receptor screen (internal data). Together with the low BBB penetration in mice, these results suggest that SEP-383103 is not CNS active.

## CONCLUSION

Ulotaront is a novel, CNS-active compound currently in Phase 3 clinical development for the treatment of schizophrenia. In contrast to all marketed antipsychotic drugs, ulotaront does not exert its efficacy via blockade of dopamine D_2_ or the serotonin 5-HT_2A_ receptors. Although the mechanism of action of ulotaront has not been fully elucidated, preclinical studies indicate that agonist activity at TAAR1 and 5-HT_1A_ receptors contributes to its efficacy.

Ulotaront has favorable chemical-physical properties, high solubility, high permeability, low binding to plasma proteins, and possesses all attributes of a Biopharmaceutics Classification System (BCS) 1 compound. The ability of ulotaront to penetrate the BBB for CNS targeting has been demonstrated in mice and rats. Ulotaront metabolism is determined by both NADPH-dependent and NADPH-independent enzymes, with CYP2D6-mediated metabolism as the major pathway though the metabolism mediated by non-CYP enzymes remains to be further investigated. Although ulotaront is an inducer of CYP2B6, and an inhibitor of CYP2D6 and OCTs, the potential for ulotaront to be a perpetrator of CYP or transporter-mediated DDIs is projected to be remote.

The biotransformation of ulotaront to the major metabolite SEP-383013 is largely determined by NADPH-independent enzymes including MAO and AO. SEP-383013 exhibits low permeability and is not well distributed to the brain, suggesting lack of CNS activity. CYP or transporter-mediated DDI potential with SEP-383103 as a perpetrator is remote.

## Supplementary Information

Below is the link to the electronic supplementary material.

Supplementary file1 (DOCX 576 KB)

Supplementary file2 (DOCX 253 KB)

## ABBREVIATIONS

ADME: Absorption, distribution, metabolism, and excretion
AO: Aldehyde oxidase
BBB: Blood-brain barrier
BCRP: Breast cancer resistance protein
BCS: Biopharmaceutics classification system
BSEP: Bile salt export pump
CYP: Cytochrome P450
CNS: Central nerve system
DDI: Drug-drug interactions
FMO: Flavin-containing monooxygenase
HEK293: Human embryonic kidney 293 cells
HLM: Human liver microsomes
MATE: Multidrug and toxin extrusion
MAO: Monoamine oxidase
NADPH: Nicotinamide adenine dinucleotide phosphate
OAT: Organic anion transporter
OATP: Organic anion transporting polypeptide
OCT: Organic cation transporter
P-gp: P-glycoprotein
PK: Pharmacokinetics
PBPK: Physiologically based pharmacokinetics
TAAR1: Trace amine-associated receptor 1
