# Supplementary material for: In Vitro ADME and Preclinical Pharmacokinetics of Ulotaront, a TAAR1/5-HT1A Receptor Agonist for the Treatment of Schizophrenia
Source: Pharm Res. 2022 Apr 28;39(5):837–50. doi: 10.1007/s11095-022-03267-1 (PMC9160101; doi:10.1007/s11095-022-03267-1)
Supplement: Supplementary file 1 — Supplementary file1 (DOCX 576 KB) [file 11095_2022_3267_MOESM1_ESM.docx]

***Supplemental:***

**Major bioanalytical LC-MS/MS methods used for *in vivo* and *in vivo* sample analysis for this work**

**A: LC-MS/UV method used for Hepatocyte and LM stability assays**

**HPLC:** Shimadzu HPLC (including pumps, autosampler, and UV detector at 254 nm)

Analytical column: Waters Atlantis T3, 150 × 4.6 mm, 5-µm

Mobile Phase A: 0.1% (v/v) formic acid in water

Mobile phase B: Acetonitrile

Flow rate: 1.0 mL/min; Split: after UV, 20% to MS, and 80% to waste

Gradient: From 0 – 3 min, MPB 5 – 10%; from 3 – 8 min, MPB 10- 50%; from 8- 10 min, MPB 50 - 100%; then back to MPB 5% and re-equilibrating for 2 mins.

**MS instrument and conditions:**

MS: Finnigan TSQ7000

Software: Xcalibur v 1.2 (acquiring data), v 2.06 (processing data)

Ionisation: Electrospray (ESI), positive ion mode

Spray voltage: 4.5 kV

Heated capillary temperature: 350 ^◦^C

Sheath gas: N_2_, 50 psi

Auxiliary gas: N_2_, 10 units

Scan range: 120 to 600 amu

Scan rate: 1 s/scan

MS/MS conditions: collision gas – Argon, 2 mTorr; collision energy 20 – 25 eV; Scan range: m/z 10 to (M+5) amu (where M is the protonated molecular ion); scan rate 1 s/scan.

**B: LC-MS/MS method used for Human Hepatocytes and Liver Microsomes, and Induction studies**

Samples were analyzed by multiple reaction monitoring LC-MS/MS methods developed at the testing facility. Analysis was performed by electrospray ionization (ESI) source coupled with Sciex (or Waters) mass spectrometer. LC system used was a Shimadzu Nexera (or Waters Acquity UPLC). Sample analysis, integration and reporting were conducted according to corresponding laboratory SOPs.

**C: LC-MS/MS method used for the transporter study**

Agilent 1290 HPLC coupled with MS/MS (Agilent 6470A) was used for sample analysis in the transporter study. The LC-MS/MS conditions are summarized below:

**D: LC-MS/MS method used for in vitro metabolism using HµRel^®^ System**

**E: LC-MS/MS methods used for phenotyping study sample assays at WuXi (XBL) Lab**

**E-1: For SEP-363856**:

UHPLC System: Shimadzu CBM-20A system controller, LC-30AD pump, SIL-30AC autosampler

Data System: Analyst^®^ 1.6.3

HPLC Column: Waters Acquity UPLC BEH C18 2.1 x 50 mm

Mobile Phase A: 0.1% Formic Acid in Water

Mobile Phase B: 0.1% Formic Acid in Acetonitrile

Flow rate: 0.60 mL/min

Gradient:

| Time (min) | 0.0 | 0.5 | 2 | 2.5 | 2.51 | 3.5 |
| --- | --- | --- | --- | --- | --- | --- |
| MPB (%) | 5 | 5 | 95 | 95 | 5 | Stop |

Mass Spectrometer: AB Sciex API 4000

Ionization Mode: ESI Positive

Scan Mode: Multiple Reaction Monitoring (MRM)

CAD: 6

CUR: 25

GS1 = GS2: 50

Ion Spray Voltage: 5.0 kV

Source Temperature: 500 °C

EP: 10 V

CXP: 10 V

Dwell time 100 ms

MS/MS parameters:

| Compound | Precursor ion (m/z) | Product ion (m/z) | DP (V) | CE (eV) |
| --- | --- | --- | --- | --- |
| SEP-363856 | 184.1 | 135.1 | 65 | 30 |
| SEP-363855-d3 | 187.2 | 135.1 | 65 | 30 |
| Labetalol | 329.2 | 162.1 | 50 | 50 |
| Tolbutamide | 271.1 | 155.3 | 50 | 25 |

**E-2: For SEP-363854:**

UPLC System: Waters UPLC

Data System: Masslynx v.4.2

HPLC Column: Waters XBridge Amide 100 x 2.1 mm, 3.5µm

Mobile Phase A: 0.1% Formic Acid in Water

Mobile Phase B: 0.1% Formic Acid in Acetonitrile

Flow rate: 0.70 mL/min

Gradient:

| Time (min) | 0.0 | 0.5 | 2 | 2.01 | 2.2 | 2.21 | 3.5 |
| --- | --- | --- | --- | --- | --- | --- | --- |
| MPB (%) | 95 | 95 | 60 | 50 | 50 | 95 | 95 |

Mass Spectrometer: Waters-TQS

Ionization Mode: ESI Positive

Scan Mode: MRM

Cone Voltage: 30 V

Desolvation gas: 800 L/hr

Cone: 150 L/hr

Ion Spray Voltage: 5.0 kV

Temperature: 500°C

EP: 10 V

Dwell time 100 ms

MS/MS parameters:

| Compound | Precursor ion (m/z) | Product ion (m/z) | CE (eV) |
| --- | --- | --- | --- |
| SEP-363854 | 170.1 | 135.1 | 20 |
| SEP-363055-d4 | 174.1 | 138.1 | 20 |

**E-3: For SEP-383103:**

HPLC System: Shimadzu CBM-20A system controller, LC-30AD pump, SIL-30AC autosampler

Data System: Analyst^®^ 1.6.3

HPLC Column: Waters ACQUITY UPLC BEH Amide 50 x 2.1 mm

Mobile Phase A: 0.2% acetic acid in water, pH 4.0

Mobile Phase B: Acetonitrile

Flow Rate: 0.60 mL/min

Gradient:

| Time (min) | 0.0 | 0.5 | 2.0 | 2.3 | 2.31 | 3.5 |
| --- | --- | --- | --- | --- | --- | --- |
| MPB (%) | 60 | 60 | 40 | 40 | 60 | stop |

Mass Spectrometer: AB Sciex API 6500

Ionization Mode: ESI Negative

Scan Mode: MRM

CAD 6

CUR 25

GS1 50

GS2 50

Ion Spray Voltage: -4.5 kV

Source Temperature: 500 °C

EP -10 V

CXP -15 V

Dwell time: 70 ms

MS/MS parameters:

| Compound | Precursor Ion  (m/z) | Product Ion  (m/z) | DP (V) | CE (eV) |
| --- | --- | --- | --- | --- |
| SEP-383103 | 183 | 137 | -35 | 11 |
| IS: SEP-376955-d4 | 187 | 140 | -35 | 11 |

**F: LC-MS/MS method used for Metabolite SEP-383103 CYP induction and inhibition**

LC-MS/MS method and conditions used for sample analysis in the SEP383103 CYP induction and inhibition study are summarized below:

**G: LC-MS/MS method used for Metabolite SEP-383103 transporter study**

LC-MS/MS method used for sample analysis in the SEP383103 transporter study are summarized below:

**H: LC-MS/MS method used for mouse plasma and brain PK measurement**

The concentrations of SEP-0363856 in mouse plasma and brain samples were determined using a liquid chromatography with tandem mass spectrometry (LC-MS/MS) based method. A protein precipitation method was used for sample preparation. For non-diluted plasma samples: An aliquot of 30 µL sample was mixed with 200 µL IS (Propranolol, 40 ng/mL in ACN). The mixture was vortexed for 5 min and centrifuged at 5800 rpm for 10 min. An aliquot of 1 µL supernatant was injected for LC-MS/MS analysis. For 10x diluted plasma samples: An aliquot of 6 µL plasma sample was mixed with 54 µL blank plasma to obtain the diluted samples, with a final sample dilution factor of 10. The extraction procedure for diluted samples was the same as those for non-diluted samples. For brain homogenate samples: The sample was homogenized with 3 volumes (v/w) of PBS. The dilution factor was 4. The samples preparation was the same as plasma samples. When calculating brain tissue concentration, a dilution factor of 4 was applied, and brain concentration is reported as “ng/g”. The instrument setup consisted of an AB Sciex API 5500 Mass Spectrometer equipped with a Waters UPLC Acquity. Separation of SEP-0363856 and the IS from potential interfering compounds in the study samples is achieved on an ACQUITY UPLC HSS T3 column by a reversed-phase gradient chromatographic method. SEP-0363856 and the IS were ionized under a positive ion spray mode and detected through the multiple-reaction monitoring (MRM) of a mass transition pair at m/z 184.2 → 135.1 for SEP-0363856 and 260.2 → 116.1 for IS, respectively. Using a plasma or brain homogenate sample volume of 30 μL, linearity was achieved in the SEP-0363856 concentration range of 1.00 to 3000 ng/mL for the IP/PO dosing group plasma samples, and 1.00-3000 ng/g for brain samples from the same PK groups.

**I: LC-MS/MS method used for Rat plasma PK measurement**

LC-MS/MS method used for rat plasma PK measurement was summarized below:

**J:** **LC-MS/MS method used for Dog plasma PK measurement**

LC-MS/MS method used for dog plasma PK measurement was summarized below:

**K: LC-MS/MS method used for Monkey plasma PK measurement**

LC-MS/MS method used for monkey plasma PK measurement was summarized below:
