# Supplementary material for: In Vitro ADME and Preclinical Pharmacokinetics of Ulotaront, a TAAR1/5-HT1A Receptor Agonist for the Treatment of Schizophrenia
Source: Pharm Res. 2022 Apr 28;39(5):837–50. doi: 10.1007/s11095-022-03267-1 (PMC9160101; doi:10.1007/s11095-022-03267-1)
Supplement: Supplementary file 2 — Supplementary file2 (DOCX 253 KB) [file 11095_2022_3267_MOESM2_ESM.docx]

**Supplemental Figures**

**Legend:**

**Figure S1:** Ulotaront disappearance following incubation with human liver microsomes at 1, 10, and 100 μM.

**Figure S2:** Inhibition of transporter-mediated uptake of metformin (10 µM). The inhibition on OCT1-mediated uptake was determined in the presence of ulotaront at 1 to 750 µM, and the inhibition on OCT2-mediated uptake was determined in the presence of ulotaront at 0.4 to 300 µM. Data are presented as mean ± S.D.

**Figure S3:** Ulotaront and SEP-383103 mouse plasma and brain PK profiles following a single oral dose of 10 mg/kg ulotaront. Concentrations are presented as mean ± S.D.

Figure S1


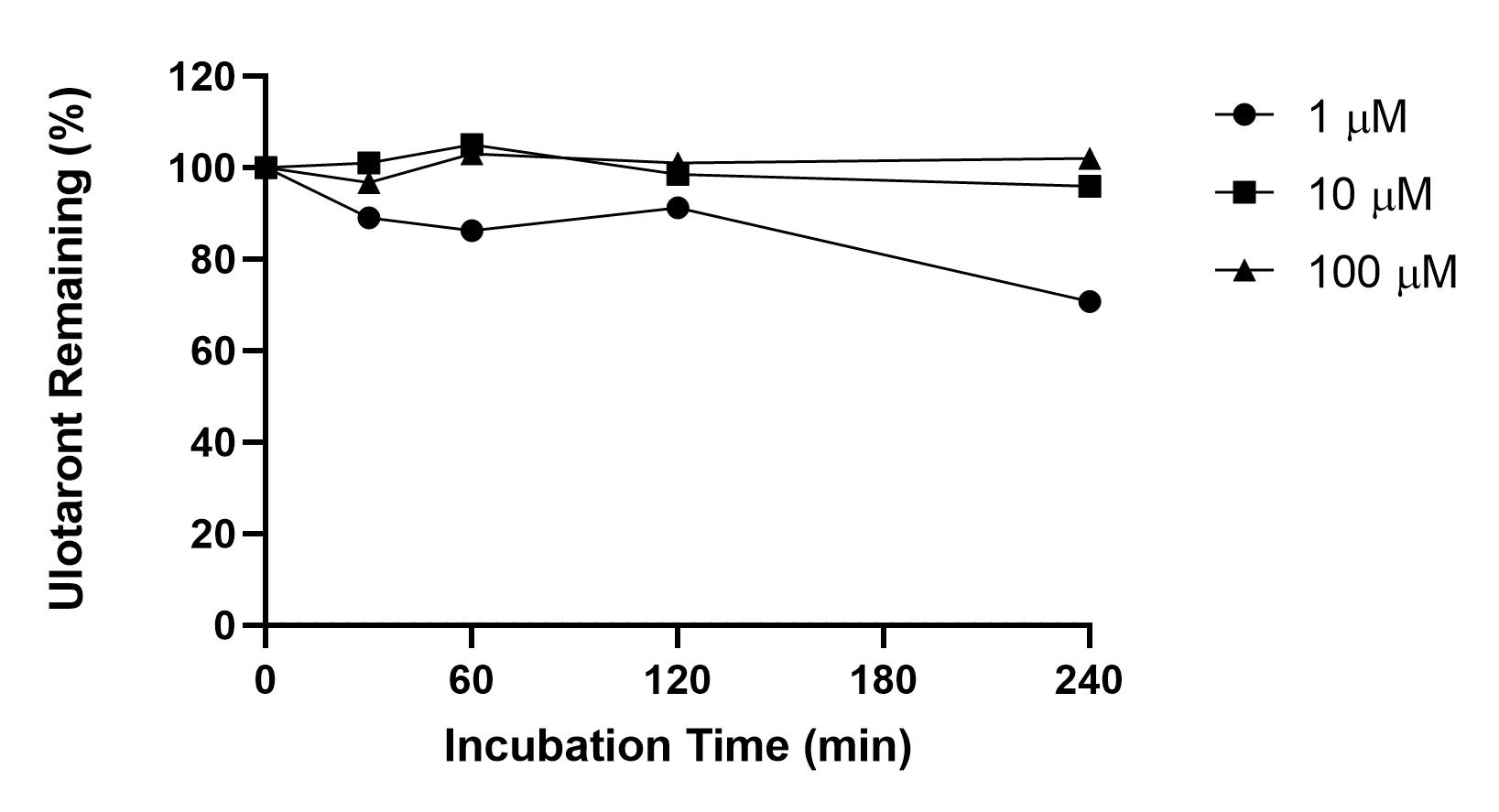


Figure S2


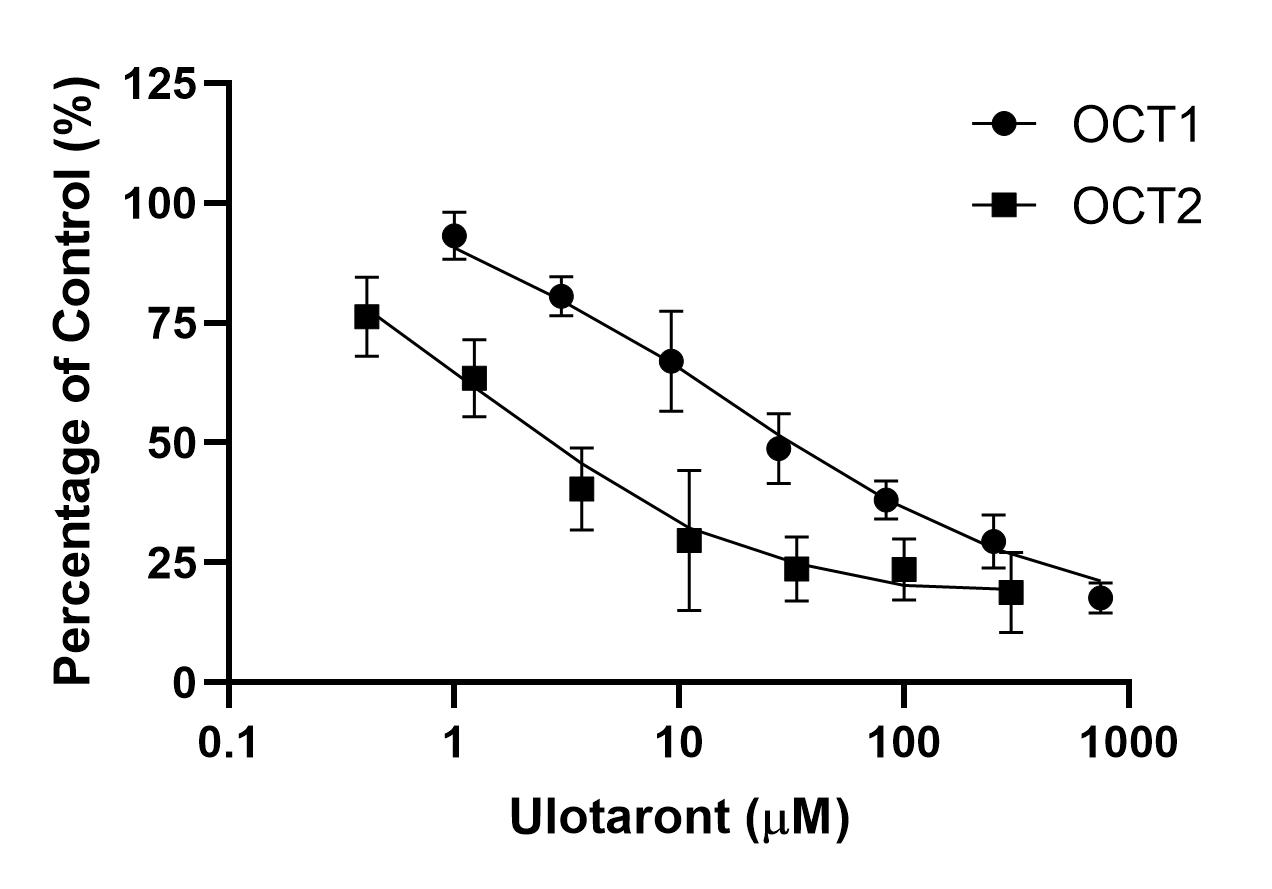


Figure S3


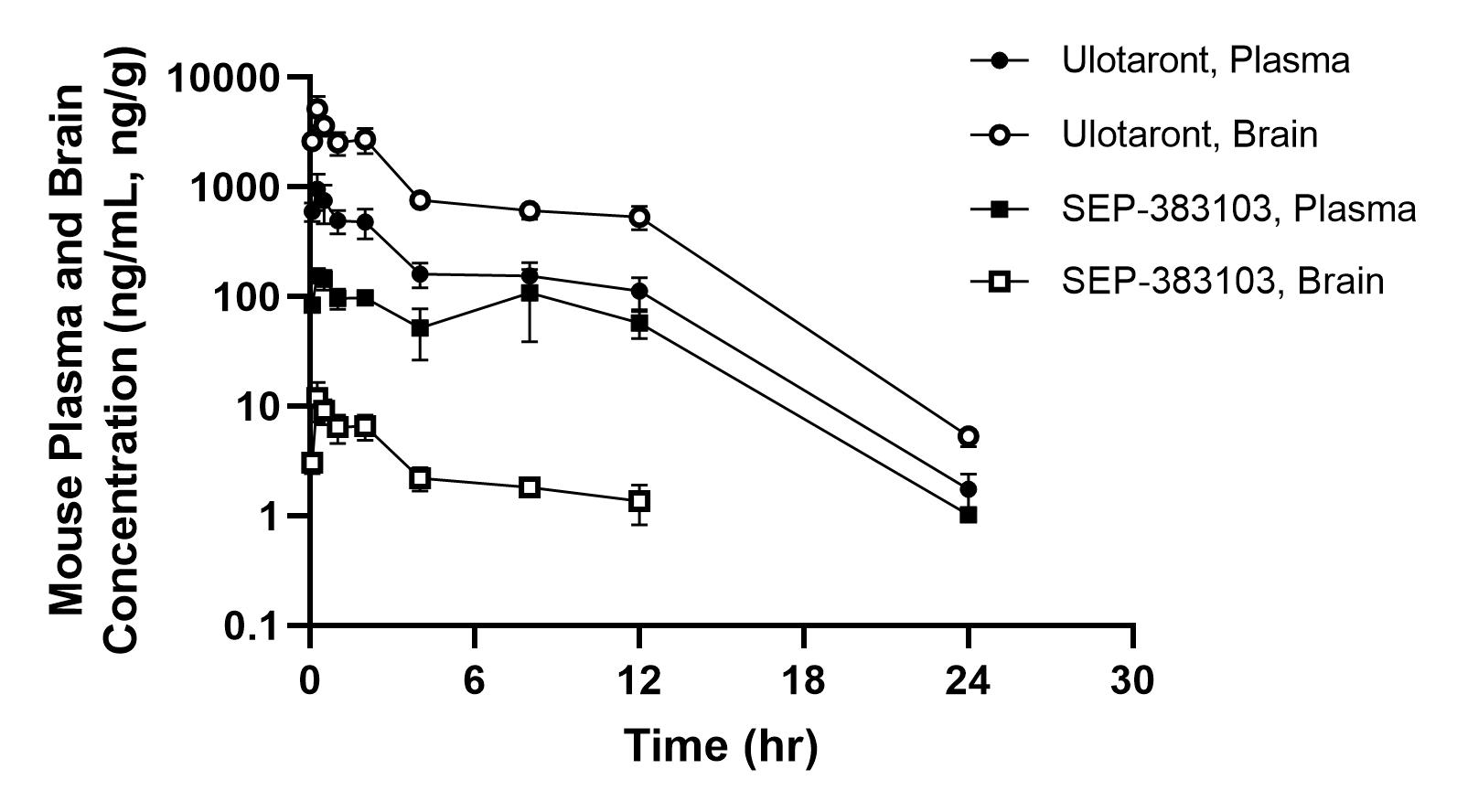


**Supplemental Tables**

| **Table S1: Probe Substrates and Prototypical Inhibitors Used for CYP Inhibition Assays** | | | |
| --- | --- | --- | --- |
| **CYP Isoform** | **Substrate** | **Direct Control Inhibitor** | **TDI Control Inhibitor** |
|  | **Ulotaront CYP Inhibition Assay** | | |
| CYP1A2 | Phenacetin | α-Naphthoflavone | Furafylline |
| CYP2A6 | Coumarin | Nicotine | 8-Methoxypsoralen |
| CYP2B6 | Efavirenz | Orphenadrine | Phencyclidine |
| CYP2C8 | Amodiaquine | Montelukast | Gemfibrozil glucuronide |
| CYP2C9 | Diclofenac | Sulfaphenazole | Tienilic acid |
| CYP2C19 | S-Mephenytoin | Modafinil | S-Fluoxetine |
| CYP2D6 | Dextromethorphan | Quinidine | Paroxetine |
| CYP2E1 | Chlorzoxazone | 4-Methylpyrazole | 3-Amino-1,2,4-triazole |
| CYP3A4/5 | Testosterone | Ketoconazole | Troleandomycin |
| CYP3A4/5 | Midazolam | Ketoconazole | Troleandomycin |
| CYP4A11 | Lauric acid | 10-IDA | 1-Aminobenzotriazole |
|  |  |  |  |
|  | **SEP-383103 CYP Inhibition Assay** | | |
| CYP1A2 | Phenacetin | 7,8-Benzoflavone | Furafylline |
| CYP2B6 | Bupropion | Ketoconazole | Ticlopidine |
| CYP2C8 | Amodiaquine | Montelukast | Gemfibrozil glucuronide |
| CYP2C9 | Diclofenac | Sulfaphenazole | Tienilic acid |
| CYP2C19 | S-Mephenytoin | S-Benzylnirvanol | S-Fluoxetine |
| CYP2D6 | Dextromethorphan | Quinidine | Paroxetine |
| CYP3A4/5 | Testosterone | Ketoconazole | Azamulin |
| CYP3A4/5 | Midazolam | Ketoconazole | Azamulin |

| **Table S2: Probe Substrates and Prototypical Inhibitors for Transporter Assays** | | |
| --- | --- | --- |
| **Transporter** | **Substrate** | **Inhibitor** |
|  | **Ulotaront Transporter Assay** | |
| OCT1 | Metformin | Verapamil |
| OCT2 | Metformin | Verapamil |
| MATE1 | Metformin | Pyrimethamine |
| MATE2K | Metformin | Pyrimethamine |
| OAT1 | Tenofovir | Probenecid |
| OAT3 | Estrone-3-sulfate | Probenecid |
| OATP1B1 | Estradiol-17-β-glucuronide | Rifampicin |
| OATP1B3 | Cholecystokinin octapeptide | Rifampicin |
| P-gp | Digoxin | Valspodar |
| BCRP | Estrone-3-sulfate | Ko143 |
| BSEP | Taurocholate | Cyclosporin A |
|  |  |  |
|  | **SEP-380103 Transporter Assay** | |
| OCT1 | Tetraethylammonium | Quinidine |
| OCT2 | Metformin | Quinidine |
| MATE1 | Tetraethylammonium | Cimetidine |
| MATE2K | Tetraethylammonium | Cimetidine |
| OAT1 | para-Aminohippurate | Probenecid |
| OAT3 | Estrone-3-sulfate | Probenecid |
| OATP1B1 | Estradiol-17-β-glucuronide | Cyclosporine A |
| OATP1B3 | Cholecystokinin octapeptide | Cyclosporine A |
| P-gp | Digoxin | Zosuquidar |
| BCRP | Estrone-3-sulfate | Ko143 |

| **Table S3: Ulotaront Plasma Protein Binding, Reb Blood Cell Partitioning, and Hepatocyte Stability** | | | | |
| --- | --- | --- | --- | --- |
| Species | fu (%) | RBC | CL_int_ (mL/min/kg) | CL_hep_ (mL/min/kg) |
| Mouse | 80.6 | 0.89 | 12.0 | 9.8 |
| Rat | 78.7 | 0.90 | 6.9 | 5.5 |
| Dog | 79.2 | 1.16 | 42.9 | 15.5 |
| Monkey | 85.9 | 1.12 | 13.4 | 8.3 |
| Human | 77.5 | 1.14 | 7.2 | 3.9 |

| **Table S4: Ulotaront Permeability across Caco-2 Monolayers** | | | | |
| --- | --- | --- | --- | --- |
| Ulotaront (µM) | Incubation Time (min) | P_app_A-B (10^-6^ cm/s) | P_app_B-A (10^-6^ cm/s) | Efflux Ratio |
| 1 | 45 | 26.7 | 29.7 | 1.1 |
|  | 90 | 23.0 | 28.7 | 1.2 |
|  | 120 | 22.0 | 29.3 | 1.3 |
| 10 | 45 | 26.0 | 28.3 | 1.1 |
|  | 90 | 24.0 | 27.7 | 1.2 |
|  | 120 | 22.7 | 28.3 | 1.3 |
| 100 | 45 | 27.3 | 29.0 | 1.1 |
|  | 90 | 25.3 | 28.3 | 1.1 |
|  | 120 | 23.7 | 28.7 | 1.2 |
